# Listen to Your Heart–Ecological Momentary Assessment of Interoceptive Accuracy, Awareness and Sensibility: A Pilot Study

**DOI:** 10.3390/ijerph18094893

**Published:** 2021-05-04

**Authors:** Inken Höller, Jana-Sophie Stenzel, Dajana Rath, Thomas Forkmann

**Affiliations:** Department of Clinical Psychology, University of Duisburg-Essen, 45141 Essen, Germany; jana-sophie.stenzel@gmx.de (J.-S.S.); dajana.rath@uni-due.de (D.R.); thomas.forkmann@uni-due.de (T.F.)

**Keywords:** interoception, heartbeat perception, EMA

## Abstract

*Background:* Interoception is a multi-facetted phenomenon including interoceptive accuracy, awareness and sensibility. Deficits in interoception have been associated with psychological distress. However, little is known about the course of interoception over time. The present study aimed at examining interoception in an ecological momentary assessment (EMA)-setting. *Methods:* A seven-day smartphone-based EMA was conducted in a community sample of sixty-one participants (age: *M* = 24.1, *SD* = 7.00, *n* = 54 female (88.5%)). To control for potential practice effects of repeated assessments during the EMA phase, participants were randomly assigned to a control (*n* = 30) and an interoception (*n* = 31) group. The latter was assessed for interoceptive accuracy, awareness and sensibility. Before and after the EMA phase, all participants were assessed for interoception in the laboratory. *Results:* Multilevel analyses revealed significant fluctuations for all three interoceptive facets, around 50% of variance was due to within-person variability. There were only practice effects for the subscale “Attention Regulation”, measuring interoceptive sensibility. *Conclusion:* The facets of interoception can be assessed in an EMA-setting. Repeated interoceptive assessments do not necessarily lead to an improvement of participants’ interoceptive abilities. It could be shown that all interoceptive facets fluctuate, which should be considered in future research.

## 1. Introduction

Interoception can be described as the ability to sense one’s own physiological condition of the body [1]. While some researchers referred to interoception as a single construct [2], others examined different facets of interoception [3]. These inconsistent terminologies and their use have been criticized by Garfinkel et al. [4], who demanded consistent and clear definitions. By examining the structure of interoception, Garfinkel, Seth, Barrett, Suzuki and Critchley [4] showed that interoception is a multi-facetted phenomenon including (at least) interoceptive accuracy, awareness and sensibility.

Interoceptive sensibility can be described as the self-evaluation of someone’s subjective interoception assessed by using self-reports [5]. Interoceptive accuracy describes someone’s actual performance in an objective interoceptive task, e.g., a heartbeat perception task, whereas interoceptive awareness comprises the metacognitive awareness of one’s own interoceptive accuracy. Garfinkel, Seth, Barrett, Suzuki and Critchley [4] showed that interoceptive awareness and sensibility could only partly predict interoceptive accuracy. All three dimensions were distinct and separable. Since there was a relationship between the facets, but only within the group of individuals with the greatest interoceptive accuracy, interoceptive accuracy has been highlighted as the core construct of interoception. The authors suggested that the relationship between the interoceptive facets is stronger for individuals with high interoceptive accuracy compared to those with low interoceptive accuracy. In a pilot study of 24 healthy students, Meessen et al. [6], in fact, demonstrated that all three facets of interoception are uncorrelated. Forkmann et al. [7] confirmed the three-dimensionality of interoception by reporting no correlations between the facets when accuracy was measured with the heartbeat perception task [8] and moderate correlations when accuracy was measured with the heartbeat discrimination task.

Research suggests that the facets of interoception seem to be associated with psychological distress, e.g., depression and anxiety [9,10,11]. For anxiety, mixed results have been reported [12]. While Garfinkel et al. [13] and Dunn et al. [14] found that interoceptive accuracy independently contributed to anxiety symptoms, other studies state that patients with panic disorder show similar or even better interoceptive accuracy [3,15,16] but worse interoceptive sensibility [3] compared to a control group. Interoceptive awareness was positively related to trait anxiety [17]. For depression, deficits in interoceptive accuracy generally seem to be associated with depressive symptoms [11,18]. A review by Eggart et al. [19] suggested a u-shaped relationship between depression and interoceptive accuracy, with largest interoceptive deficits coinciding with moderate depression severity.

Only recently, it has been proposed that interoceptive deficits might also be related to suicidal ideation and behavior [20]. Interoceptive sensibility measured with the self-report measure Multidimensional Assessment of Interoceptive Awareness (MAIA) [5] and assessed with the subscale “interoceptive deficits” from the Eating Disorder Inventory (EDI-3) [21] was negatively associated with current suicidal ideation and past suicide attempts [22,23,24]. MAIA scores could differentiate between suicide attempters, suicidal ideators and a control group [25]. The EDI even differentiated recent and distant suicide attempters [20,26]. There are only two studies available so far that investigated interoceptive accuracy (i.e., the objective performance in detecting body sensations) in relation to suicidal ideation or behavior. Results showed no differences in interoceptive accuracy (but in interoceptive sensibility) for participants with compared to those without suicidal ideation [27] and lower heartbeat perception accuracy in suicide attempters compared to non-attempters [28]. However, there has been no study investigating associations between interoceptive awareness and suicidality [29].

Regarding the assessment of interoceptive sensibility, there are two critical points: on the one hand, many studies assessed interoceptive sensibility with a subscale of the EDI [20,26], which was originally developed for participants with eating disorders and assesses interoception in relation to food intake and the gastrointestinal system [21]. Thus, it appears at least questionable whether conclusions based on EDI-data can be generalized to patients with no eating but other mental disorders. On the other hand, in self-report questionnaires, interoceptive sensibility was usually assessed retrospectively and the timeframe respondents are asked to refer to was not specified. For example, the MAIA refers to “the general daily life” [5], while the EDI refers to how often each statement applies with no time frame at all [21]. It has been argued that questionnaire-based retrospective assessments are compromised by memory bias and a lack of ecological validity [30].

It is unclear whether interoception is best understood as being state-like or trait-like. There is evidence from few studies suggesting within-person change in interoception across time. A study by Wittkamp et al. [31] using latent state-trait analysis of interoceptive accuracy assessments on three consecutive measurement occasions showed that 40% of variance in one single interoceptive accuracy measurement could be explained by trait, whereas 27% was traced to effects of situation and person-situation interactions–suggesting some variability in interoceptive accuracy over time. Some further evidence comes from studies that investigated whether facets of interoception could be trained. Studies showed that interoceptive awareness trained by daily practices of “Body Scans” and “Breath Meditation” [32] and accuracy trained by daily “Body Scans” over eight weeks [33] and by contingent cardiac feedback [34] could be improved, suggesting that interoception can generally be affected by situational or behavioral manipulations (i.e., training), which implies a certain temporal variability. Only interoceptive sensibility appeared not to be affected by “Body Scan” interventions [33]. It is unclear, however, whether the mere repeated execution of the various interoception measurements already results in a practice effect as the studies mentioned above explicitly trained the facets through interventions. It is also unclear how the facets of interoception behave over time and whether they are measurable over short time intervals of minutes or hours.

A viable alternative assessment method that promises the possibility to overcome memory bias, lack of ecological validity and allows for the assessment of within-person variation across short time frames, is Ecological Momentary Assessment (EMA). EMA refers to the repeated sampling of subjects’ current behaviors and experiences in real time and in their natural environments [35], for example, via smartphones [36,37]. There is empirical evidence on the within-person variation and temporal trajectories of clinical variables such as suicidal ideation, depression and anxiety [38,39,40,41]. Although as noted above, interoception has been shown to be related to all these clinical variables, to date, we know virtually nothing about the temporal course of the facets of interoception across short intervals of minutes or hours.

While interoceptive sensibility is usually assessed via self-report, which can rather easily be adopted to the EMA-setting (such as already implemented for, e.g., suicidal ideation or negative affect [42]), the assessment of interoceptive accuracy and awareness is more challenging, since it requires the collection of both self-reported information and the number of heartbeats in given time-frames.

Therefore, the aim of this study was to measure interoceptive accuracy, awareness and sensibility using EMA. Since the facets of interoception have never been investigated in such a study design, this study should be treated as a pilot study. The main goal of this study was to test the general feasibility of such a study design and to find out whether the facets of interoception are subject to intraindividual fluctuations. Because of the novelty of the design and potential test burden associated with repeated EMA, we abstained from including patients with mental disorders but decided to aim for a non-clinical sample to prove the study concept. Based on prior findings on interoception, we hypothesized that (a) all three facets of interoception fluctuate over time. Additionally, we hypothesized that (b) there will be no practice effect for the facets of interoception through mere interoceptive task repetition, since positive practice effects have only been shown for targeted interventions and not solely for mere task repetition.

## 2. Materials and Methods

### 2.1. Participants

The final sample consisted of *n* = 61 participants aged between 18 and 51 years (*M* = 24.21, *SD* = 6.99). Participants were eligible if they were at least 18 years old, had sufficient knowledge of the German language, had no current mental disorder and did not abuse drugs or alcohol. In order to keep the study comparable to other studies assessing interoception [4,43,44], participants needed to be physically healthy and showing a body mass index (BMI) between 18.5 and 24, since it has been shown that obesity affects the ability to detect feedback of cardiovascular functions and, thereby, influences an individual’s interoceptive abilities [45]. Additionally, they should neither take medication influencing the cardiovascular system nor participate in competitive or endurance sports more than three times a week. Fifty-four participants were female (88.5%). Most participants were unmarried (*n* = 57; 93.4%) and working (*n* = 39; 63.9%). Only ten participants lived alone (16.4%). Twenty participants (32.8%) reported a mental disorder in their past, assessed with the short version of the diagnostic interview for mental disorders (Mini-DIPS) [46]. Three participants (4.9%) reported a suicide attempt in their lifetime. Participants were randomly assigned to one of two groups (interoception vs. control group). The randomization was conducted with www.randomizer.org by generating a random order of the numbers 1 and 2 (1 = interoception group, 2 = control group). Participants were assigned consecutively to these groups in the randomized order after examining the eligibility criteria. Both groups completed the same EMA with only one difference: the control group (*n* = 30) did not complete interoception tasks during the EMA phase of the study, while the interoception group (*n* = 31) was assessed for all three facets of interoception via EMA.

### 2.2. Procedure

Participants were recruited between June and December 2019 via postings in Facebook groups as well as with flyers at the University of Duisburg-Essen and other places open to the public (such as public library). Participants who were interested in participating got in touch with the study team via e-mail. The study team then contacted those possible participants for a telephone interview. The telephone interview was conducted to check in and exclusion criteria, such as sufficient knowledge of the German language, no current mental disorder, no drug or alcohol abuse and a BMI between 18.5 and 24. Since this was a pilot study and the main goal was to examine interoception in an EMA-setting, we aimed for participants without a current mental disorder to keep the test burden low. Participants were asked whether they were currently, diagnosed with a mental disorder or were receiving treatment for a mental disorder. In case of current mental disorders, participants were immediately excluded from study participation. For assessing the BMI, participants were asked for their height and their weight. Then, the BMI was calculated. If the BMI was below 18.5 or over 24, participants were excluded from the study. When participants met the eligibility criteria, they were invited to our lab. Prior to the assessments, participants were informed about the purpose of the study, the voluntary nature of their participation, data storage and security. They gave written informed consent before participating. The study was approved by the responsible Ethic Committee of the University of Duisburg-Essen and was in accordance with the Declaration of Helsinki [47]. For their participation, participants received 60 EUR or five hours of study credit and 10 EUR. Participants’ recruitment first included a telephone interview. The study included three main assessments (baseline, EMA, post), which are described in detail in the following.

#### 2.2.1. Baseline Assessment

**Figure 1:**
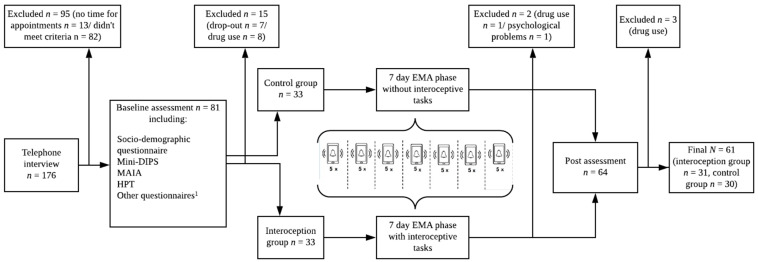
Study Procedure. ^1^ Other questionnaires, which have not been analyzed for this study; Mini-DIPS = structured clinical interview for *ICD* disorders; MAIA = Multidimensional Assessment of Interoceptive Awareness; HPT = Heartbeat Perception Task; EMA = Ecological Momentary Assessment. Baseline assessment was identical to post assessment.

Participants underwent a baseline assessment (T0) in our lab including a structured clinical interview (Mini-DIPS) [46] on mental disorders to verify the participants’ report in the prior telephone interview that they were currently not suffering from a mental disorder. The Mini-DIPS was conducted by researchers who had at least a Bachelor Degree in psychology and were familiar with the ICD-10 classification system of mental diseases [48]. Additionally, those researchers had received a training in conducting the Mini-DIPS prior to the start of this study. In case a current mental disorder was diagnosed, participants were excluded right away and were informed about the diagnoses and treatment options at the outpatient clinic for mental health at the local university and additional contacts to get help. All participants without a current mental disorder and/or a mental disorder in the past filled out questionnaires. Additionally, they conducted a heartbeat perception task (pre-HPT) in our lab to assess participants’ interoceptive accuracy and awareness. If they reported drug use in the sociodemographic questionnaire, which they had not mentioned before, they were excluded (see Figure 1).

#### 2.2.2. 7-Day EMA

After the baseline assessment, participants were introduced to the EMA method (i.e., overview of the app, charging the phone, carrying the phone at all times). They received an android study smartphone, used for data collection via the app movisensXS, v1.4.8 (movisens GmbH, Karlsruhe, Germany) The study smartphone could only be used for this app; all other applications were blocked. Participants in the interoception group were additionally equipped with a wearable smartwatch (Polar A370; Polar Electro GmbH, Büttelborn, Germany) and were instructed to wear the smartwatch from 8 am to 8 pm for seven days. Additionally, they were reminded to do so every morning via the app movisensXS. Participants then underwent a seven-day EMA with five assessments per day outside of the lab, resulting in a maximum of 35 assessments per participant. These five assessments per day were randomly presented between 8 am and 8 pm with at least two hours between two measurements. A short beep announced the beginning of an assessment. Assessments were randomized in the time of their occurrence throughout the day. Participants could postpone (15 min) or completely reject a prompt if they were not able to answer. In case the phone battery ran below 20%, participants received a notification to charge the phone. Since the single assessments were short, the overall time of assessments was <30 min per day. Individual results were uploaded from the smartphone to a webserver via mobile data directly after completion of each assessment, allowing the research team to check compliance rates. Each participant received three text messages for motivational purposes or in case their compliance rate dropped below 80% during the course of the entire EMA phase. All participants received the same assessments and where prompted 5 times per day for 7 days. Participants in the control group answered 20 items including several constructs such as mood, context and suicidal ideation. For participants in the interoception group, each assessment additionally included 8 items assessing interoceptive sensibility and an EMA-HPT. Interoception items and the EMA-HPT task were designed specifically for this study by the authors and the movisensXS support team. Participants in the interoception group were instructed not to perform excessive activities before or during the single assessments. All participants were provided with information of a German helpline and also had the possibility to contact the study staff through the messenger option within the app in case of technical questions or in case they felt burdened. Messages were checked at a regular daily basis. No participant reported to feel burdened through the assessments.

#### 2.2.3. Post Assessment

After the EMA phase, participants were invited to a post assessment inclusively returning the study smartphone and the smartwatch. Participants had to participate in the post assessment within the latest of 14 days after the EMA phase. The post assessment (T2) took place in the same lab as T0 and was identical to the baseline assessment except for the Mini-DIPS and the sociodemographic questionnaire, which were excluded in the post assessment. Participants received several questionnaires and conducted the post-HPT. Figure 1 provides an overview of the procedure.

### 2.3. Measures

Measures relevant for the aims of the present study will be described in detail. Further information on the other measures can be found elsewhere [49].

#### 2.3.1. Baseline and Post Assessment Measures

##### Multidimensional Assessment of Interoceptive Awareness (MAIA)

The questionnaire contains 32 items rated on a six-point scale ranging from “never” (0) to “always” (5) including eight subscales. Mean subscale scores are calculated for the subscales Noticing, Not-Distracting, Not-Worrying, Attention Regulation, Emotional Awareness, Self-Regulation, Body Listening and Trusting. Higher scores indicate higher interoceptive sensibility. Internal consistencies (Cronbach’s α) at T0 for the single scales varied between 0.55 and 0.89, Cronbach’s α at T2 varied between 0.64 and 0.93. The MAIA was applied before and after the EMA phase resulting in pre- and post-data [50].

##### Pre- and Post-Heartbeat Perception Task (HPT)

For the heartbeat perception task, participants were seated in our lab and provided with electrodes. The actual heart rate was recorded via electrocardiogram (ECG) using a BIOPAC MP150 (Biopac, Santa Barbara, CA, USA). Participants were instructed to sit quietly during the entire experiment. First, a five minute baseline was conducted. Then, participants received the instruction of the heartbeat perception task on a computer screen. The heartbeat perception task was programmed with Presentation^®^ software v18.0 (Neurobehavioral Systems, Inc., Berkeley, CA, USA). In line with the Mental Tracking Method described by Schandry [8], participants were asked to silently count their heartbeats in randomized intervals of 25, 35 and 45 s for ten trials. The beginning and end of the counting phases were indicated by a start and a stop tone. Participants were instructed to not take their pulse or to use any manipulations enabling their counting. Additionally, participants had to enter the number of their counted heartbeats and were asked for a confidence judgement of their performance ranging from 0 to 100% (“How sure are you on a scale from 0 to 100 percent that your estimation is correct?”. There was a 60 s break between each trial. Participants did not receive any information about the length of the intervals or their performance. After five trials, participants received a break of 5 min for assessing another baseline. For the ten trials, an accuracy score (pre- and post-HPT score) was derived according to Schandry [8]:

(1) $$HPS=\frac{1}{10}*\;\sum \left(1-\frac{\left|recorded\;heartbeats-perceived\;heartbeats\right|}{recorded\;heartbeats}\right)$$

The score indicates interoceptive accuracy and ranges from 0 to 1 with lower scores indicating poor heartbeat perception. Negative values are possible in case the number of the perceived heartbeats exceeds 200% of the recorded heartbeats [7]. Interoceptive awareness was calculated by computing the differences between judgments of confidence and the respective HPT score. Since the HPT score ranged from 0 to 1 and the confidence judgment ranged from 0 to 100, the variable of the HPT score was transformed by multiplying the HPT score with 100. Then, we subtracted the HPT score from the awareness score. A positive value as a result of this calculation means that a person overestimates his/her own performance, a negative value indicates an underestimation of one’s own performance. A value around 0 indicates a good awareness.

#### 2.3.2. Items Used during the EMA Phase

##### EMA-MAIA

In order to capture interoceptive sensibility entirely by use of MAIA items during EMA, factor loadings of the MAIA items from validation studies were compared [5,51]. One item with the highest factor loading from each of the eight MAIA subscales [5,51] was selected, resulting in a total of eight items. Items were adapted to the moment-to-moment design by adding “At the moment” to the beginning of the respective item and adjusting the item wording to the moment in order to capture the real-time character and the moment-to-moment variability (e.g., Noticing: “At this moment, I notice where in my body I am comfortable; Body Trust: At this moment I feel my body is a safe place” (Supplementary Materials available: detailed list of all items used during EMA). All eight items had to be rated on a five-point Likert scale ranging from “not at all” (1) to “very strong” (5).

##### EMA-HPT

One trial of the HPT task was used in each assessment. Participants were asked to silently count their heartbeat for a time interval lasting either 25, 35 or 45 s. The time interval was marked by two beep tones from the smartphone indicating the start and the end of the interval (“In the following task you will hear a short beep. After a while a second beep will follow. Your task is to count your heartbeat as accurately as possible in the time between the two beeps. After the second beep, click on the check mark in the upper right corner to enter the number of heartbeats you have counted.”). Then, participants were requested to enter the number of counted heartbeats in the MovisensXS app: “Please enter the exact number of your counted heartbeats.”. The task was completed with the answer of participants to how sure they were about their estimation on a scale from 0 to 100% (Confidence Judgement; “How sure are you that the number of your counted heartbeats matches your actual heartbeats? (in percent)”). The actual heart rate of participants was recorded with the Polar A370. Polar devices have shown good validity and reliability in measuring heartrate [52,53,54]. The Polar A370 shows a more reasonable accuracy than other Polar devices [55] and allows a wrist-based heart rate measurement using a technology called optical heart rate monitoring (OHR). Heartrate data were collected every second by the device. The device provides time-stamped HR data allowing matching of the data with the EMA-HPT trial after transferring the data of the Polar A370 to the Polar FlowSync desktop app v3.0.0.1337 (Polar Electro GmbH, Büttelborn, Germany). For the analyses, data of the time intervals of the EMA-HPT were extracted and time-matched. EMA-HPT scores were calculated with these heart rate measurements following the formula of Schandry [8] mentioned above. Interoceptive awareness during EMA was calculated as for T0 and T2.

### 2.4. Statistical Analyses

Since, to the best of our knowledge, interoception has never been assessed using smartphone-based EMA before, we will first report descriptive statistics of the three facets of interoception measured via EMA and in the laboratory at baseline and post assessment. Especially the assessment of interoceptive accuracy via EMA outside of the laboratory is a new method. We expected that baseline assessments in the laboratory and the day 1 EMA-based assessments of interoceptive accuracy should give similar results, since those assessments were conducted within short time intervals. The same applies to the last day (day 7) of EMA and the post assessment. *T*-tests for dependent samples were implemented to test differences in means between pre-HPT and day 1 EMA-HPT, as well as post-HPT and day 7 EMA-HPT. No differences would indicate validity of interoceptive accuracy assessed via EMA.

#### 2.4.1. EMA-Data (Interoception Group Only)

For the analyses regarding hypothesis a), only the EMA data of the interoception group were used. The dataset consisted of 35 (assessments on level 1) × 31 (persons on level 2) = 1085 observations. On average, participants completed 79.6% of the assessments. Missing data at level 1 were handled with pairwise deletion for each correlation pair and for each beep per person. Through this pairwise deletion, it is possible to include this person for all further beeps and for all further correlation pairs. Because of the nested structure of the data, multilevel analyses using the statistical software HLM v7.03 (Scientific Software International, Inc., Chapel Hill, NC, USA) and RStudio v1.3.1093 (R Team, Boston, MA, USA) software, including the package ggplot2 [56], were conducted.

For examining fluctuations, intercept-only models were calculated for all three facets of interoception. Variance components of these intercept-only models were used to compute intra-class correlations (ICC) as an indicator of the proportion of variance explained by the different levels [57]. For assessing variability across time, Mean Squared Successive Differences (MSSD) were calculated. The MSSD can be described as the sum score of the squared differences between two measurements in time series and, therefore, represents point-to-point variability. Higher values indicate higher fluctuation [58]. To illustrate those possible fluctuations of the facets of interoception, we plotted the fluctuations across all 35 assessments points for each participant and separately for each interoceptive facet.

#### 2.4.2. Baseline and Post Assessment Data

For the second hypothesis of the study, stating that there is no practice effect for the facets of interoception through repetition of interoceptive tasks, data from the entire sample were used. Mixed factorial ANOVAs and simple ANOVAs were calculated in SPSS v26 software (IBM Deutschland GmbH, Ehningen, Germany) to reveal differences in interoceptive accuracy, awareness and sensibility (pre-HPT vs. post-HPT) between groups (control group vs. interoception group) and between assessments (baseline vs. post assessment).

## 3. Results

Descriptive statistics of all study variables can be found in Table 1. *T*-tests for dependent samples revealed no significant difference between mean interoceptive accuracy measured in the laboratory at T0 (pre-HPT: *M* = 0.59; *SD* = 0.09) and mean interoceptive accuracy at day 1 measured via EMA (day 1 HPT-EMA: *M* = 0.59; *SD* = 0.27; *t*(28) = −0.064, *p* = 0.95). In addition, there was no difference between mean interoceptive accuracy measured in the laboratory at T2 (post-HPT: *M* = 0.60, *SD* = 0.11) and mean interoceptive accuracy measured at day 7 via EMA (day 7 HPT-EMA: *M* = 0.60, *SD* = 0.23; *t*(27) = 0.138, *p* = 0.89). This suggests that results of EMA-based assessments of interoceptive accuracy (HPT-EMA) do not differ from assessments of interoceptive accuracy in the laboratory, supporting the validity of assessing interoceptive accuracy based on EMA outside the laboratory.

### 3.1. Short-Term Variability of Interoception during EMA

**Figure 2:**
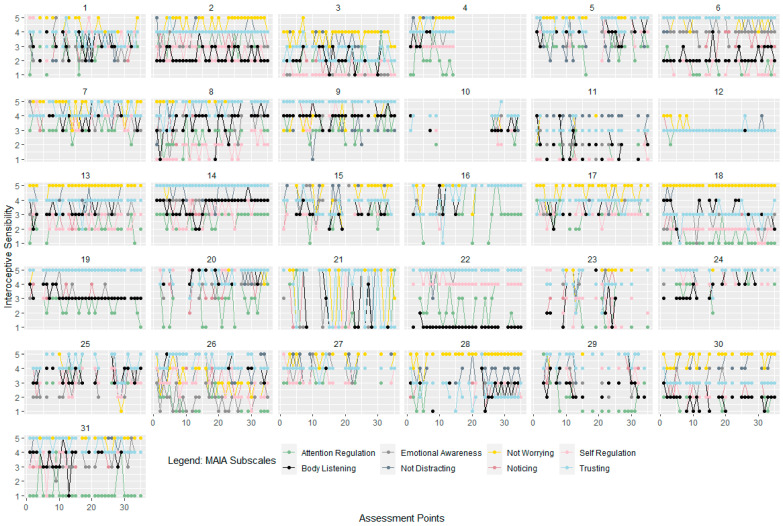
Interoceptive Sensibility. Trajectories of the subscales of the Multidimensional Assessment of Interoceptive Awareness for each of the 31 participants during EMA. Assessment points range from 1 to 35 over a time period of 7 days and interoceptive sensibility ranges from 1 to 5 (total score of each of the subscales).

**Figure 3:**
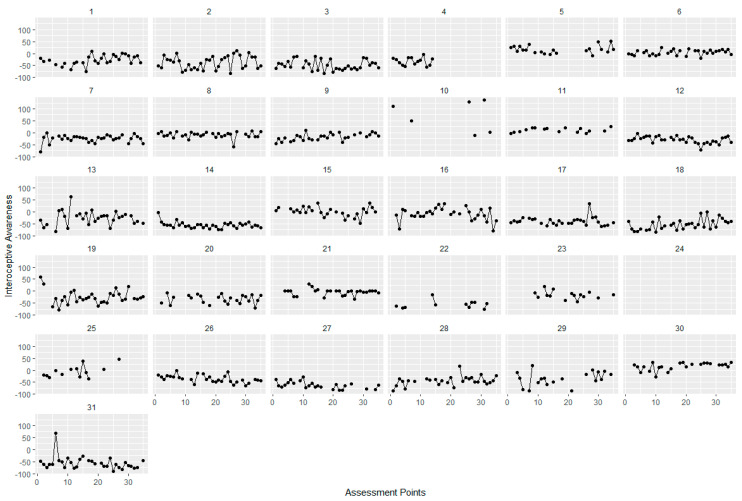
Interoceptive Awareness. Trajectories of interoceptive awareness for each of the 31 participants during EMA. Assessment points range from 1 to 35 over a time period of 7 days, interoceptive awareness ranges from −100 to 100.

**Figure 4:**
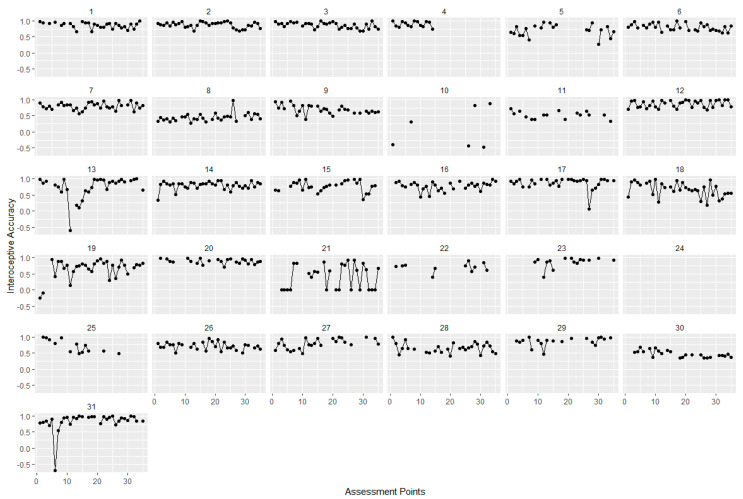
Interoceptive Accuracy (EMA-HPT). Trajectories of interoceptive accuracy measured with the heartbeat perception task for each of the 31 participants during EMA. Assessment points range from 1 to 35 over a time period of 7 days, interoceptive accuracy ranges from −1 to 1.

For interoceptive awareness, 37% of the variance was accounted for by within-person variability (over time, see Table 1). For the EMA-HPT score (as a measure of interoceptive accuracy), ICCs indicated that 58% of variance was due to within-person variability (over time). Between 38% and 62% of variance in the MAIA subscales as a measure of interoceptive sensibility during EMA was due to within-person variability (over time), depending on the subscale. For “Emotional Awareness” and “Self-Regulation” the proportion of variance due to within-person variability was 38%, while for “Attention Regulation”, 62% of variance was due to within-person variability. Additionally, MSSDs, as can be seen in Table 1, demonstrated a wide range, indicating large differences between individuals in intra-individual variability across time. For the MAIA subscales during EMA, attention regulation seemed to have the highest point-to-point variability in comparison to the other scales. Figure 2, Figure 3 and Figure 4 show the individual trajectories of interoception during EMA for each of the facets separately over 35 assessments for the participants of the interoception group, sorted by subject ID.

### 3.2. Practice Effects of Interoception from Baseline to Post Assessment

The mixed factorial ANOVA did not show a statistically significant interaction between time (baseline assessment vs. post assessment) and group (interoception group vs. control group) for interoceptive accuracy (pre- vs. post-HPT). There was also no significant main effect for time or group. For interoceptive awareness, there was neither a statistically significant interaction between time and group nor a significant main effect for time or group (see Table 2).

For interoceptive sensibility results of each MAIA scale is reported separately. For four of the subscales, there were significant results. There was a significant main effect of time (*F*(1, 59) = 5.17, *p* < 0.05) for the scale “Noticing”. Independently of group, participants improved from the baseline to the post assessment (*M*_BaselineInteroception_ = 3.31, *SD*_BaselineInteroception_ = 0.79, *M*_BaselineControl_ = 3.26, *SD*_BaselineControl_ = 0.81, *M*_PostInteroception_ = 3.65, *SD*_PostInteroception_ = 0.74, *M*_PostControl_ = 3.39, *SD*_PostControl_ = 0.68).

For “Attention Regulation”, there was a significant interaction effect of time*group (*F*(1, 59) = 3.96, *p* < 0.05). Simple ANOVAs showed that there were only significant differences between the interoception and the control group at the post assessment (*F*(1, 59) = 6.65, *p* < 0.05) but not at the baseline assessment (*F*(1,59) = 1.18, *p* = 0.28). At the post assessment, participants in the interoception group showed significantly higher scores in “Attention Regulation” than participants in the control group (*M*_PostInteroception_ = 3.21, *SD*_PostInteroception_ = 0.83, *M*_PostControl_ = 2.68, *SD*_PostControl_ = 0.78).

In regard to “Emotional Awareness”, there was only a significant main effect of time (*F*(1, 59) = 5.48, *p* < 0.05). Independently of group, participants improved from the baseline to the post assessment in their self-reported “Emotional Awareness” (*M*_BaselineInteroception_ = 3.44, *SD*_BaselineInteroception_ = 0.98, *M*_BaselineControl_ = 3.42, *SD*_BaselineControl_ = 0.91, *M*_PostInteroception_ = 3.76, *SD*_PostInteroception_ = 0.76, *M*_PostControl_ = 3.58, *SD*_PostControl_ = 0.79).

In regard to “Body Listening”, there was a significant main effect of time (*F*(1,59) = 4.04, *p* < 0.05). Independently of group, participants improved from the baseline to the post assessment in their self-reported ability to listen to their body (*M*_BaselineInteroception_ = 2.76, *SD*_BaselineInteroception_ = 0.99, *M*_BaselineControl_ = 2.56, *SD*_BaselineControl_ = 0.99, *M*_PostInteroception_ = 3.18, *SD*_PostInteroception_ = 0.93, *M*_PostControl_ = 2.62, *SD*_PostControl_ = 1.06).

## 4. Discussion

The aim of the current study was to assess interoception in an EMA-setting to examine its temporal course. We hypothesized that the facets of interoception fluctuate (a) and that there is no practice effect for the facets of interoception based on repetition of interoceptive tasks (b).

### 4.1. Fluctuations of Interoception during EMA

Given the current findings, the first hypothesis that interoceptive accuracy, awareness and sensibility fluctuate could be confirmed. Around 50% of the variance in all three facets of interoception was due to within-person variability. It could also be confirmed that this variability differed considerably between individuals, which complements findings of Wittkamp, Bertsch, Vögele and Schulz [31].

The present results that interceptive accuracy, awareness and sensibility fluctuate across time are in line with results of EMA-based studies measuring clinical variables. Similar to the facets of interoception, it has been shown that depression, anxiety, and suicidal ideation fluctuate across time [38,39,40,41]. All these variables have been shown to be related to interoception [13,18,20]. Thus, results of this study call for future studies investigating the longitudinal association between clinical variables such as depression or suicidal ideation and interoception across time. The high temporal resolution of EMA facilitates the identification of differential relations between clinical variables and improves the understanding of an individual patient’s dynamic symptom change. Since compliance was excellent in the present study and in prior studies investigating clinical samples [42], future EMA-studies assessing interoception in clinical samples are warranted. Results of this study support the general feasibility of assessing interoception in EMA-studies.

### 4.2. Practice Effects of Interoception from Baseline to Post Assessment

Regarding the second hypothesis of different facets of interoception not being improved by repetitive interoceptive tasks, our findings were mixed. Most importantly, for interoceptive accuracy as well as awareness, there were no significant practice effects. At first glance, this result appears to be contrary to findings of Bornemann, Herbert, Mehling and Singer [32] and Fischer, Messner and Pollatos [33], who showed that interoceptive accuracy and awareness could be improved by training. However, their trainings contained interventions such as the daily practice of “Body Scan” and contingent cardiac feedback during the HPT, which were both specifically targeted at improving interoception, whereas in the present study, no specific training was applied but only the effect of repeated practice was examined. In the interoception group, only the interoception tasks were presented repeatedly compared to the control group. Participants in this study did not receive feedback and, therefore, could not notice whether they improved or not. Conclusively, interoceptive tasks, especially the HPT as a performance task, can be repeated and assessed multiple times without noticeable practice effects. Fluctuations and interindividual differences appear to be interpretable and should not be traced to practice effects only.

For future studies with a similar setup, it would be interesting to examine how such highly repeated contingent cardiac feedback in short time intervals over several days would influence the interoceptive accuracy and awareness. This is especially interesting in regard to clinical practice. Interoceptive EMA interventions would allow patients to autonomously train repetitively by themselves wherever they are, which could also potentially improve their self-efficacy. In the light of recent studies, the training of interoceptive skills could have a positive impact on e.g., depression [59] and chronic worry [60] and could, therefore, be of high benefit in clinical practice.

For interoceptive sensibility, the MAIA scale “Attention Regulation” showed significant practice effects. Participants in the interoception group showed significantly higher scores at the post assessment than participants in the control group. Since “Attention Regulation” seems to be improvable through practice, one could speculate whether a targeted training of “Attention Regelation” might have beneficial effects on mental and/or physical health. However, it is important to keep in mind that in the current study participants could have only improved in their self-reported “Attention Regulation” because they were forced to give more attention to themselves than usually through the repetitive EMA.

Since there were also significant main effects of time for the scales “Noticing”, “Emotional Awareness” and “Body Listening”, participants also improved in those scales measuring interoceptive sensibility independently from group. Thus, improvements in interoceptive sensibility should not only be attributed to repeated assessments of interoception in EMA. Instead, only one repetition of these measurements (T0–T2) seems sufficient to increase interoceptive sensibility, at least for certain scales. However, results could be different in a clinical sample and need replication before further conclusions should be derived.

### 4.3. Strengths and Limitations

The results of the present study should be appreciated in the light of some strengths and limitations. The major strength of the study was being the first to assess the facets of interoception in EMA and, therefore, providing important findings about how the facets of interoception behave over time. Since there were no substantial practice effects for accuracy and awareness, it seems to be valid to measure the facets of interoception repeatedly. Furthermore, *t*-tests did not reveal differences in means between the assessments of interoceptive accuracy in EMA and at the baseline and post assessment supporting the validity of the assessment of interoceptive accuracy outside a laboratory setting. Additionally, for the baseline and post assessment in the laboratory, heartbeats were recorded via electrocardiogram (ECG) using a BIOPAC MP150.

As already stated, the current study is the first to assess interoception in an EMA design. Therefore, the primary aim of the study was to show the feasibility of assessing interoception within EMA and to investigate its temporal course. Since EMA is associated with some test burden in daily life, we deliberately abstained from including participants with mental disorders. We were able to show, in this study, the principal feasibility of the assessment strategy and the temporal fluctuation of interoception. In our view, this pattern of results warrants further investigation, ideally in clinical populations.

The first limitation of this study is that resting heart rate of participants was only assessed before the pre- and post-HPT at the baseline and post assessment. During the EMA phase, we did not control for arousal, which is negatively correlated with interoceptive accuracy [61]. However, participants were familiar with the HPT task and practiced this task in our laboratory. When giving instructions to participants for the EMA phase, we explicitly reminded participants not to perform excessive activities before or during the EMA phase. We also asked participants during EMA what they had been doing before, where they were and if anything noteworthy had happened since the last assessment. There was no statistical control for context effects in the analyses of this study, since answers to these questions were heterogeneous and did not allow quantitative analyses. We did calculate the average heart beats per minute of our sample with *M* = 84 and *SD* = 10 (min. = 65, max. = 112). For future studies, one possibility could be to use bolus infusions of isoproterenol, a non-selective beta adrenergic agonist, which elicits rapid increases in heart rate and has been shown to overcome a major limitation of HPTs [62]. Another possibility would be to instruct participants additionally at the beginning of each assessment to sit down and rest for 5 min before starting the assessment. However, compliance for such an extended approach might be low, since it would take longer for the participant.

Second, the HPT [8] seems to be influenced by non-interoceptive processes [63] and interoceptive accuracy scores derived from it are potentially problematic [64]. Even though different psychological processes such as emotion regulation capacities [65] and decision-making [66] have been related to interoceptive accuracy scores derived from the HPT highlighting its role in psychological research, Zamariola et al. [64] propose four criticisms, with which Ainley et al. [67] insistently disagreed arguing that three of the four criticisms are not valid (for more detailed information see: Ainley et al. [67]).

(1) Zamariola, Maurage, Luminet and Corneille [64] state that interoceptive accuracy measured with the HPT depends on the error of participants’ undercounting of their perceived heartbeats due to their beliefs about their heart rate. Ainley, Tsakiris, Pollatos, Schulz and Herbert [67] counter that participants’ beliefs do not explain why participants would rate their heart rates lower than they are and, thereby, their beliefs would not particularly explain their possible undercounting, which is contradictory to the first criticism.
(2) The number of recorded heartbeats and the number of perceived heartbeats does not correlate (in Zamariola, Maurage, Luminet and Corneille’s data [64]). However, Ainley, Tsakiris, Pollatos, Schulz and Herbert [67] found that Zamariola, Maurage, Luminet and Corneille [64] made this assumption due to arithmetic misunderstanding, which disproves this second criticism.
(3) Zamariola, Maurage, Luminet and Corneille [64] state that a measure for interoceptive accuracy should not depend on heart condition. However, this should actually be treated in favor of the HPT’s construct validity, since it is clear that the perception of interoceptive signals is depending on one’s specific physiology [67].
(4) Last but not least, there seems to be a tendency to poorer performance on the longer trials of the HPT. This statement is rejected by Ainley, Tsakiris, Pollatos, Schulz and Herbert [67] arguing that mean recorded heart rates significantly differed between the three lengths of the trials in the data of Zamariola, Maurage, Luminet and Corneille [64], which is in contrast to their assumption that the heart rate is constant across intervals and the poorer performance for longer trials is traced to participants’ undercounting.

In conclusion, the HPT is significantly connected to the activity of interoceptive neural networks, which has been shown in multiple studies [68,69], the HPT has been shown to provide information about the associations between an individual’s interoceptive accuracy and psychological distress such as depression [11], anxiety [13] and even suicidality [28] and most of its critical points have been disproved. Thus, we feel secure about the use of this measure for assessing interoceptive accuracy.

Third, participants had to complete the post assessment to the latest 14 days after the EMA phase. This was a wide time frame and should be kept shorter in future studies. It could be possible that practice effects already vanish within short periods of time.

Fourth, the results could also be influences by the high percentage (88.5%) of female participants, since men seem to be better in perceiving interoceptive processes [70,71]. However, Pennebaker and Roberts [72] suggest that those sex differences vanish in a non-laboratory setting. For future studies, it would be interesting to examine sex differences in interoception as well as hormonal changes and their influence on interoception during EMA, which is not a typical laboratory setting.

## 5. Conclusions

The present study is the first to assess interoceptive accuracy, awareness and sensibility repeatedly across time by means of ecological momentary assessments. Results support the general validity of such a measurement approach and revealed considerable within-person variability for all three facets. Practice effects were not found for interoceptive accuracy and awareness, but should be considered for interoceptive sensibility. The results call for replication in clinical samples. Increasing general scientific interest in research in interoception and a growing body of evidence suggesting its potential link to mental illnesses [73,74,75,76] call for further investigations. Recent research suggests that symptoms of mental disorders such as depression, anxiety, or suicidal ideation show substantial within-person variance and fluctuate across time [38,39,40,41]. Thus, future EMA-studies should consider investigating the prospective relations between interoception and symptoms of mental disorders to shed further light on its complex relations and potential interactions.

## Figures and Tables

**Table 1:** Descriptive statistics and variability indices of baseline, post and ecological momentary assessments.

Construct and Items	T0 ^1^				T2 ^1^				EMA ^2^				MSSD (EMA Items)				ICC
	*M*	*SD*	Min.	Max	*M*	*SD*	Min	Max	*M*	*SD*	Min.	Max	*M*	*SD*	Min	Max	
Interoceptive Accuracy (HPT)	0.58	0.10	0.26	0.79	0.60	0.11	0.24	0.82	0.72	0.18	0.12	0.90	0.05	0.05	0.01	0.27	0.42
Interoceptive Awawareness	−7.62	23.62	−52.28	50.56	−7.75	23.25	−58.20	56.03	−21.99	28.15	−62.41	69.06	720.62	493.06	79.85	2006.43	0.63
Interoceptive Sensibility ^3^																	
Noticing	3.28	0.79	1.50	5.00	3.52	0.72	1.00	5.00	3.49	0.73	1.97	5.00	0.72	1.08	0.00	5.82	0.56
Not-Distracting	2.24	0.87	0.33	4.33	2.16	0.83	0.67	4.33	4.17	0.61	3.06	4.97	0.87	0.87	0.03	4.79	0.42
Not-Worrying	2.61	0.96	0.33	5.00	2.55	0.94	0.33	5.00	4.46	0.62	2.70	5.00	0.61	0.90	0.00	4.79	0.52
Attention Regulation	2.96	0.75	1.14	4.43	2.95	0.84	1.14	4.71	2.60	0.69	1.29	3.95	1.26	0.91	0.06	4.39	0.38
Emotional Awareness	3.43	0.94	1.20	5.00	3.67	0.77	1.40	5.00	3.40	0.85	1.09	4.97	0.78	0.97	0.00	5.44	0.62
Self-Regulation	2.72	1.07	0.25	5.00	2.77	1.14	0.00	5.00	3.07	0.92	1.20	4.85	0.76	1.07	0.00	6.11	0.62
Body Listening	2.66	0.99	0.00	5.00	2.90	1.02	0.67	5.00	3.28	0.83	1.03	4.58	0.95	1.42	0.00	7.00	0.56
Trusting	3.89	0.93	0.33	5.00	4.05	0.83	1.33	5.00	4.08	0.74	2.50	5.00	0.65	1.35	0.00	7.52	0.61

HPT = Heart Beat Perception Task; ^1^ *n* = 61 (total sample including interoception and control group), T0 = Baseline assessment, T2 = Post assessment, ^2^ *n* = 31 (interoception group only), EMA = Ecological Momentary Assessment, *M* = mean, *SD* = standard deviation, Min. = minimum, Max. = maximum, MSSD = mean squared successive difference, ICC = intraclass correlation; ^3^ Interoceptive sensibility was assessed with the subscale of the Multidimensional Assessment of Interoceptive Awareness, during EMA one item for each subscale was chosen.

**Table 2:** Results of mixed factorial ANOVAs.

Effects		F	*p*	η^2^
Interoceptive Accuracy				
Interaction effect	Time * Group	0.26	0.614	.004
Within-subject effect	Time	1.74	0.193	.029
Between-subject effect	Group	1.13	0.292	.019
Interoceptive Awawareness				
Interaction effect	Time * Group	0.15	0.697	0.003
Within-subject effect	Time	0.00	0.952	0.000
Between-subject effect	Group	0.05	0.828	0.001
Interoceptive Sensibility: MAIA scales				
Interaction effect	Time (Noticing ^1^) * Group	0.98	0.327	0.016
Within-subject effect	Time (Noticing ^1^)	5.17	0.027 *	0.081
Between-subject effect	Group	0.85	0.359	0.014
Interaction effect	Time (Not-Distracting ^1^) * Group	0.01	0.937	0.000
Within-subject effect	Time (Not-Distracting^1^)	0.96	0.331	0.016
Between-subject effect	Group	3.42	0.070	0.90
Interaction effect	Time (Not-Worrying ^1^) * Group	0.15	0.703	0.002
Within-subject effect	Time (Not-Worrying ^1^)	0.40	0.532	0.007
Between-subject effect	Group	1.81	0.184	0.030
Interaction effect	Time (Attention Regulation ^1^) * Group	3.96	0.051 *	0.063
Within-subject effect	Time (Attention Regulation^1^)	0.06	0.808	0.001
Between-subject effect	Group	4.13	0.047 *	0.065
Interaction effect	Time (Emotional Awareness ^1^) * Group	0.62	0.434	0.010
Within-subject effect	Time (Emotional Awareness ^1^)	5.48	0.023 *	0.085
Between-subject effect	Group	0.26	0.612	0.004
Interaction effect	Time (Self-Regulation ^1^) * Group	1.98	0.165	0.032
Within-subject effect	Time (Self-Regulation ^1^)	0.23	0.636	0.004
Between-subject effect	Group	2.76	0.102	0.045
Interaction effect	Time (Body Listening ^1^) * Group	2.25	0.139	0.037
Within-subject effect	Time (Body Listening ^1^)	4.04	0.049 *	0.064
Between-subject effect	Group	2.97	0.090	0.048
Interaction effect	Time (Trusting ^1^) * Group	1.29	0.261	0.021
Within-subject effect	Time (Trusting ^1^)	3.09	0.084	0.050
Between-subject effect	Group	1.30	0.260	0.021

Within-subject effect = Time (Performance in baseline assessment and post assessment of the respective measurement); between-subject factor = Group (interoception group (*n* = 31) and control group (*n* = 30), ^1^ MAIA (= Multidimensional Assessment of Interoceptive Awareness) scales, * *p* < 0.05.

## Data Availability

All relevant data are reported within the paper and are available from the corresponding author upon reasonable request.

## Supplementary Materials

The following are available online at https://www.mdpi.com/article/10.3390/ijerph18094893/s1, Study Material: List of all items used during the EMA.
