# Supplementary material for: Listen to Your Heart–Ecological Momentary Assessment of Interoceptive Accuracy, Awareness and Sensibility: A Pilot Study
Source: Int J Environ Res Public Health. 2021 May 4;18(9):4893. doi: 10.3390/ijerph18094893 (PMC8124337; doi:10.3390/ijerph18094893)
Supplement: Supplementary file 1 [file ijerph-18-04893-s001.zip › ijerph-1179555-supplementary.pdf]

Supplementary Materials

Construct	Items in German:	Items in English:
Morning reminder	Guten Morgen! Wir würden Sie nun bitten, die Trainingsphase mit der Fitnessuhr zu starten, damit die Aufzeichnung Ihrer Herzrate beginnen kann. Vielen Dank!	Good morning! We would now like to ask you to start the training phase with your fitness watch so the recording of your heart rate can begin. Thank you very much!
Depression	Im Moment fühle ich mich traurig.	At this moment I feel sad.
	Im Moment fühle ich mich niedergeschlagen.	At this moment I feel downhearted
Positive affect	Im Moment fühle ich mich fröhlich.	At this moment I feel cheerful.
	Im Moment fühle ich mich glücklich.	At this moment I feel happy.
Defeat	Im Moment habe ich das Gefühl, dass ich aufgegeben habe.	At this moment I feel that there is no fight left in me.
	Im Moment habe ich das Gefühl, ganz unten angekommen zu sein.	At this moment I feel that I have sunk to the bottom of the ladder.
Entrapment	Im Moment fühle ich mich wie in einem tiefen Loch, aus dem ich nicht hinauskann.	At this moment I feel like I am in a deep hole I cannot get out of.
	Im Moment kann ich keinen Weg aus meiner momentanen Situation sehen.	At this moment I can see no way out of my current situation .
Active suicidal ideation	Im Moment möchte ich sterben.	At this moment I want to die.
	Im Moment denke ich darüber nach, mir das Leben zu nehmen.	At this moment I think about taking my life,
Passive suicidal ideation	Im Moment habe ich das Gefühl, dass das Leben nichts lebenswert ist.	At this moment life is not worth living for me.
	Im Moment gibt es für mich mehr Gründe zu sterben, als zu leben.	At this moment there are more reasons to die than to live for me.
Acquired Capability: Pain tolerance	Im Moment könnte ich sehr viel (körperlichen) Schmerz aushalten.	At this moment I could take a lot of (physical) pain.
Acquired Capability: Fearlessness of death	Im Moment habe ich überhaupt keine Angst vor dem Tod.	At this moment I have no fear of death at all.
Acquired Capability: Explicit	Im Moment könnte ich mich umbringen, wenn ich wollte.	At this moment I could kill myself if I wanted to.

HPT (25, 35 or 45 seconds)	In der folgenden Aufgabe werden Sie einen kurzen Piepton hören. Nach einer Weile folgt ein zweiter Piepton. Ihre Aufgabe ist es, in dem Zeitraum zwischen den beiden Tönen Ihren Herzschlag möglichst genau mitzuzählen. Nachdem der zweite Ton erfolgt ist, klicken Sie bitte auf den Haken oben rechts, um danach die Anzahl ihrer gezählten Herzschläge eingeben zu können.	In the following task you will hear a short beep. After a while a second beep will follow. Your task is to count your heartbeat as accurately as possible in the time between the two beeps. After the second beep, click on the check mark in the upper right corner to enter the number of heartbeats you have counted.
Interoceptive accuracy	Bitte geben Sie die genaue Anzahl Ihrer gezählten Herzschläge an.	Please enter the exact number of your counted heartbeats.
Interoceptive awareness	Wie sicher sind Sie sich, dass die Anzahl Ihrer gezählten Herzschläge mit Ihren tatsächlichen Herzschlägen übereinstimmt? (in Prozent)	How sure are you that the number of your counted heartbeats matches your actual heartbeats? (in percent)
Interoceptive sensibility: Noticing	Im Moment merke ich, wo in meinem Körper in mich wohlfühle.	At this moment I notice where in my body I am comfortable.
Interoceptive sensibility: Not distracting	Im Moment lenke ich mich von unangenehmen Empfindungen ab.	At this moment I distract myself from sensations of discomfort.
Interoceptive sensibility: Not worrying	Im Moment fühle ich mich unwohl und mache mir Sorgen, dass irgendetwas nicht stimmt.	At this moment I feel discomfort and start to worry that something is wrong.
Interoceptive sensibility: Attention regulation	Im Moment ist um mich eine Menge los aber ich kann dennoch meiner inneren Körperempfindungen gewahr bleiben.	At this moment there is a lot going on around me but I can maintain awareness of my inner bodily sensations.
Interoceptive sensibility: Emotional awareness	Im Moment bin ich glücklich oder fröhlich und merke wie sich mein Körper anfühlt.	At this moment I notice how my body changes when I feel happy or joyful.
Interoceptive sensibility: Self-regulation	Im Moment kann ich meinen Atem dazu nutzen, innere Spannungen abzubauen.	At this moment I can use my breath to reduce tension.
Interoceptive sensibility: Listen to the body	Im Moment höre ich auf meinen Körper, um zu erkennen was zu tun ist.	At this moment I listen to my body to inform me about what to do.
Interoceptive sensibility: Body trust	Im Moment empfinde ich meinen Körper als einen sicheren Ort.	At this moment I feel my body is a safe place.
Context: Environment	Wo befanden Sie sich gerade als das Signal kam?	Where were you just when the signal for this assessment came?

Context: Employment	Was haben Sie gerade gemacht als das Signal zur Befragung kam?	What were you doing when the before the assessment started?
Context: Society	Waren Sie zu dem Zeitpunkt als das Signal zur Befragung kam in Gesellschaft?	Were you in company at the time the assessment started?
Context: Effort	Waren Sie zu dem Zeitpunkt als das Signal zur Befragung kam körperlich angestrengt?	Were you physically strained at the time of the assessment?
Context: Special features	Geben Sie bitte hier an, falls seit der letzten Messung irgendetwas Besonderes vorgefallen ist (z. B. Erfahrungen, Aktivitäten, Ereignisse, usw. – diese können sowohl positiv als auch negativ sein). Falls nichts vorgefallen ist, tragen Sie bitte einfach eine Null ein.	Please indicate here if anything special has happened since the last assessment (e.g. experiences, activities, events, etc. - these can be both positive and negative). If nothing has happened, please simply enter a zero.
Evening reminder	Guten Abend! Für heute haben Sie es geschafft. Wir bitten Sie darum, die Trainingsphase auf Ihrer Fitnessuhr nun zu beenden. Bitte prüfen Sie außerdem den Akkustand des Geräts und laden Sie die Uhr über Nacht auf. Vielen Dank!	Good evening! You are done for today. We kindly ask you to stop the training phase on your fitness watch now. Please also check the battery status of the watch and the smartphone and charge the devices overnight. Thank you very much!
